# Surface Tension Directed Fluidic Self-Assembly of Semiconductor Chips across Length Scales and Material Boundaries

**DOI:** 10.3390/mi7040054

**Published:** 2016-03-28

**Authors:** Shantonu Biswas, Mahsa Mozafari, Thomas Stauden, Heiko O. Jacobs

**Affiliations:** Fachgebiet Nanotechnologie, Technische Universität Ilmenau, Gustav-Kirchhoff-Strasse 1, D-98693 Ilmenau, Germany; shantonu.biswas@tu-ilmenau.de (S.B.); m.mozafari@tu-ilmenau.de (M.M.); thomas.stauden@tu-ilmenau.de (T.S.)

**Keywords:** fluidic self assembly, macroelectronics, printable electronics

## Abstract

This publication provides an overview and discusses some challenges of surface tension directed fluidic self-assembly of semiconductor chips which are transported in a liquid medium. The discussion is limited to surface tension directed self-assembly where the capture, alignment, and electrical connection process is driven by the surface free energy of molten solder bumps where the authors have made a contribution. The general context is to develop a massively parallel and scalable assembly process to overcome some of the limitations of current robotic pick and place and serial wire bonding concepts. The following parts will be discussed: (2) Single-step assembly of LED arrays containing a repetition of a single component type; (3) Multi-step assembly of more than one component type adding a sequence and geometrical shape confinement to the basic concept to build more complex structures; demonstrators contain (3.1) self-packaging surface mount devices, and (3.2) multi-chip assemblies with unique angular orientation. Subsequently, measures are discussed (4) to enable the assembly of microscopic chips (10 μm–1 mm); a different transport method is introduced; demonstrators include the assembly of photovoltaic modules containing microscopic silicon tiles. Finally, (5) the extension to enable large area assembly is presented; a first reel-to-reel assembly machine is realized; the machine is applied to the field of solid state lighting and the emerging field of stretchable electronics which requires the assembly and electrical connection of semiconductor devices over exceedingly large area substrates.

## 1. Introduction

One of the main obstacles to the continuation of scaling of modern electronic systems can be found in the minimal component size that can be assembled and electrically connected effectively. Current technology for chip-scale packaging and assembly relies on robotic pick-and-place. For example, a high-end assembly system (e.g., FCM 10,000, Muehlbauer AG, Roding, Germany) is able to assemble about 8000 chips per hour with a placement accuracy of 30 μm considering a typical chip size of 1 mm. Microscopic chips and higher alignment accuracy decrease the throughput dramatically [1,2] and can generally not be assembled effectively. Moreover, robotic pick-and-place faces challenges in cases where the substrates are not rigid and perfectly planar. However, the next generation of electronics is going to be mechanically flexible [3,4,5,6] and in some cases stretchable [7,8,9,10,11]. An alternative assembly process that enables assembly on any substrate (hard, soft, flexible, stretchable) and topology (curved, convex, concave, *etc.*) are in high demand. The ideal assembly platform should be highly parallel and support assembly and interconnection of a wider range of component sizes and topologies. There may not be one ideal solution to fit all applications, and application-specific adaptations are likely going to be required to, for example, fit a desired substrate, component size, or assembly density.

Researchers have been constantly seeking an alternative to serial robotic pick-and-place on a single component basis, and several highly-parallel assembly techniques have been proposed. Two novel assembly options have emerged. The first option uses the concepts of parallel transfer [12,13,14,15,16,17] to transfer chip and nanoscopic device segments from a donor wafer to a target substrate in large quantities. In general, the transfer printing usually contains three main steps: (i) fabricating devices on a donor wafer with a sacrificial layer; (ii) pickup using an intermediate elastomeric transfer stamp; and (iii) transfer of the components to a final substrate.

Transfer processes are challenged when it comes to the redistribution of chips, assembly on curved topologies, alignment to soft or flexible substrates with non-uniformity in localized expansion coefficients. Furthermore, the assembly of large quantities of disparate parts from several donor wafers is more challenging. Moreover, parallel wafer scale transfer does not support the standard of binning which is conventionally used. Binning addresses the issue of defective device segments, which are removed from the donor wafer. Maps of defective units are created and only good ones are assembled into a final product.

The second option is to utilize concepts of directed and engineered self-assembly [18,19,20,21,22,23,24,25,26,27,28,29,30,31,32,33,34,35,36,37,38,39,40,41,42,43,44,45,46,47]. In contrast to parallel transfer, where functional devices are transferred from a donor wafer to a target substrate, maintaining orientation and functional density, self-assembly is advantageous because it can distribute chips over larger areas. For example, a binned container full of semiconductor dies/chiplets of a certain type and quality can be redistributed and assembled at precise locations on a substrate at any desired pitch or functional density using methods of directed self-assembly.

Directed self-assembly is a fairly old technique, and this publication is not meant to be a review of the entire field of directed self-assembly. For example, the concept of surface tension has been used extensively before as a method for alignment [18] or to fold structures out of plane [19]. Instead, the focus is to give insight into a particular approach of surface tension directed self-assembly which uses molten solder bumps to capture, align, and electrically connect semiconductor chips on a common substrate to enable a new kind of surface mount and packaging applications. So, the field is chip assembly.

Considering this field of massively-parallel chip assembly using self-assembly, Smith *et al.* [20] pioneered an approach that used gravity and complementary-shaped openings to capture chips from a suspension and to assemble them onto a surface in an aligned fashion. The approach was well-received and inspired research into alternative fluidic assembly methods. The field grew rapidly and different groups demonstrated the successful application of fluidic self-assembly of chips at various scales [20,21,22,23,24,25,26,27,28,29,30,31,32,33,34,35,36,37,38,39,40]. Reported methods use geometric shape recognition in combination with gravity [20,21], hydrophobic and hydrophilic forces [22,23,24], surface tension in combination with solder bumps [25,26,27], magnetic [28,48] or electrophoretic forces [29,30,31], and capillary self-transport [49], to name a few. However, considering assembly yield and the minimal component size, not all of the published methods were promising. So far, only two of the reported methods achieved yields that are on par or exceed those of robotic pick and place. The first is the original method by Smith [20], who used gravity in combination with complementary 3D shape opening to trap the chips. The second method builds on the original method by Jacobs [32], who used gravity for the introduction of the components in combination with the reduction of interfacial free energy of molten solder bumps for the assembly and alignment of the components. This method continues to be relevant, since it can capture, assemble, and electrically connect chips in a massively parallel manner over a wider range of component sizes than previously reported. The yield is high and many application-specific adaptations exist. The goal of this manuscript is to present some of the adaptations, demonstrate applications, and discuss the remaining challenges.

## 2. Single-Step Assembly of LED Arrays Containing a Repetition of a Single Component Type

**Figure 1:**
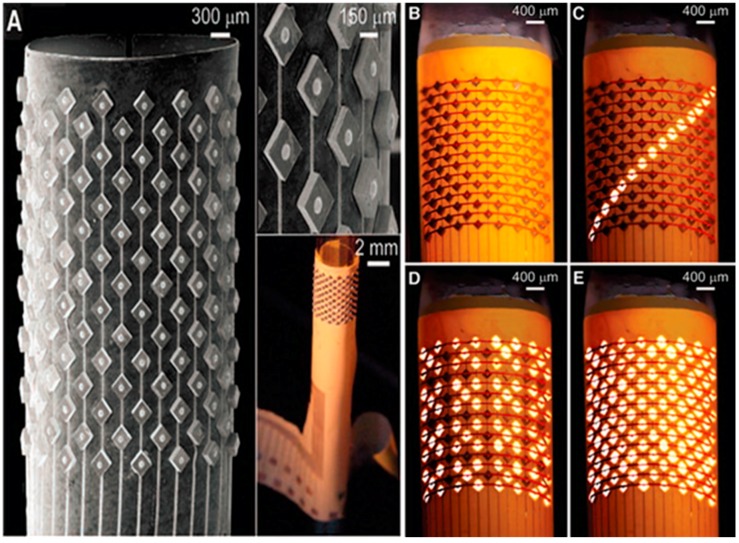
Micrographs of cylindrical display assembled in two self-driven assembly steps: Patterned self-assembly of LEDs onto a bottom electrode and self-alignment of a top electrode. (**A**) SEM and optical images of LEDs that assembled into a regular array. (**B**) Photograph of the display after self-alignment of the top electrode. (**C**–**E**) Photographs of the operating display segment. Reprinted with permission from [32].

The second process, self-assembly using gravity in combination with surface tension, was originally used to fabricate a cylindrical display [32]. Here, surface tension directed self-assembly was used to assemble and electrically connect LED chips onto a polyimide substrate. The reduction of surface tension of molten solder bumps in contact with the metallic chip surface was used as a mechanism to drive the self-assembly, and consequently to cause the electrical connection. The contact pad of the LED chips attached to the molten solder bump during the process. Figure 1 presents images of a display segment that contains 113 LEDs in eight columns and seven rows.

## 3. Multi-Step Assembly of More than One Component Type Adding a Sequence and Geometrical Shape Confinement to the Basic Concept to Build More Complex Structures

### 3.1. Self-Packaging Surface Mount Devices

**Figure 2:**
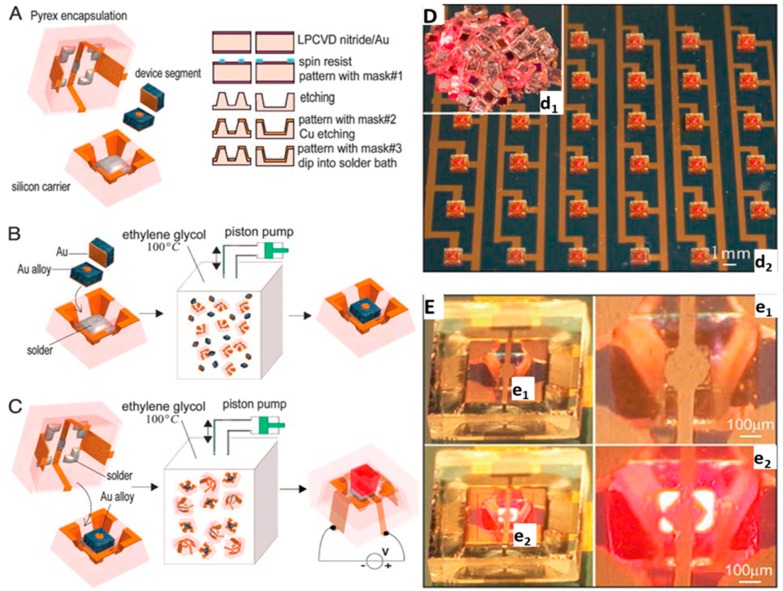
Fabrication strategy to assemble and package integrated semiconductor devices by sequential-shape-and-solder-directed self-assembly (SSSDSA). (**A**) Layout of the self-assembly components: carrier, device, and encapsulation unit that are fabricated by surface micromachining and etching. The illustrated device segment is an LED that has two contacts: a small circular anode on the front, and a large square cathode covering the back. The silicon carrier has a solder-coated area in a tapered opening to host a single semiconductor device segment. The encapsulation unit has five solder-coated copper areas inside a tapered opening to connect to corresponding contact pads on the device and carrier. Images of Chip-on-carrier assembly (**B**) and chip encapsulation (**C**) in an ethylene glycol solution at a temperature of 100 °C, where the solder is liquid. The components are agitated using a piston pump and self-assemble and form a three-dimensional circuit path between device layers that allows testing in a surface-mount device configuration. (**D**,**E**) Testing of LED chips that have been assembled and packaged by SSSDSA. (**D**) Photograph of a cluster of 200 encapsulated devices that were assembled by the self-assembly process (**d_1_**) and test of an array of LEDs that are mounted by hand on a printed circuit board (**d_2_**). (**E**) Close-up photographs of the LED assemblies in the OFF (**e_1_**) and ON (**e_2_**) state, visualizing the formation of three-dimensional circuit paths between the different device layers and the printed circuit board. From [36], copyright (2004) National Academy of Sciences, USA.

In subsequent research, the liquid-solder-directed-self-assembly process was extended and applied to the packaging of semiconductor chips, as illustrated in Figure 2. Conceptually, three different design elements were combined: (i) sequential self-assembly; (ii) geometrical shape recognition; and (iii) liquid-solder-directed-self-assembly; hence the name sequential-shape-and-solder-directed-self-assembly (SSSDSA) [36].

In the assembly process, three components were used: a Si carrier, a semiconductor device segment, and a Pyrex glass encapsulation unit (Corning, Corning, NY, USA). As presented in Figure 2A–C, each Si carrier was fabricated to accommodate one single LED chip inside a cavity. The solder coated area at the bottom of the cavity acts as the binding site for LED during the self-assembly process and as electrical contact to operate the device. The assembly process was carried out in two steps: first, chip-on-carrier assembly, and second, chip encapsulation. During the first assembly step, the molten solder wets the gold-coated cathode of the LED, and the LED chip attaches to the carrier as a result. The second assembly step encapsulates the structure and completes the electrical connections. The encapsulation unit was made out of Pyrex glass. Each unit had a 200 μm-deep tapered opening in the center. Each opening had five exposed solder-coated areas to bind to the correspondingly-shaped gold-coated side on the LED carrier. Figure 2D,E present images of assembled devices following the SSSDSA process. In the assembly process, 3000 LED chips were mixed with 600 carrier units inside the assembly medium, and the authors managed to obtain a 100% assembly yield in two minutes. For the second process, the excess LEDs were removed and the encapsulation process was performed with a yield of 97%, which means 3% of the LEDs did not function properly.

### 3.2. Multi-Chip Assemblies with Unique Angular Orientation

Geometrical shape recognition and solder directed fluidic self-assembly allow the assembling of micro-components with high accuracy and efficiency. However, angular orientation of assembled components remained a challenge. Angular orientation of devices is important because chips, packaging, or optical elements have to be assembled on the substrate with the right angular alignment to enable device performance or contact pad registration.

Zheng *et al*. reported a self-assembly process that allows the integration and electrical connection of semiconductor dies on surfaces with single-angular orientation and contact-pad-registration [39]. The key building blocks of the process were two-element docking sites on the substrate, which contained alignment pedestals and solder-coated areas similar to printed circuit board technology. The pedestals were made out of Si or photoresist. The elements were designed according to the size and shape of the components in the desired location where components needed to be assembled. These two elements, alignment pedestals and solder-coated areas, enable unique angular-alignment control of the components. Different components with different dimensions or functionality could be assembled using subsequent batch transfer process on different docking sites on a substrate. The assembly process was different from the precise complementary shape recognition technique because the process integrated components on a surface and the docking site acted as a chaperone to guide the assembly process, which prevented defects. During the assembly process, the components were agitated and driven to the solder-coated area. The assembly happened only when the correct angular pre-orientation condition was met.

**Figure 3:**
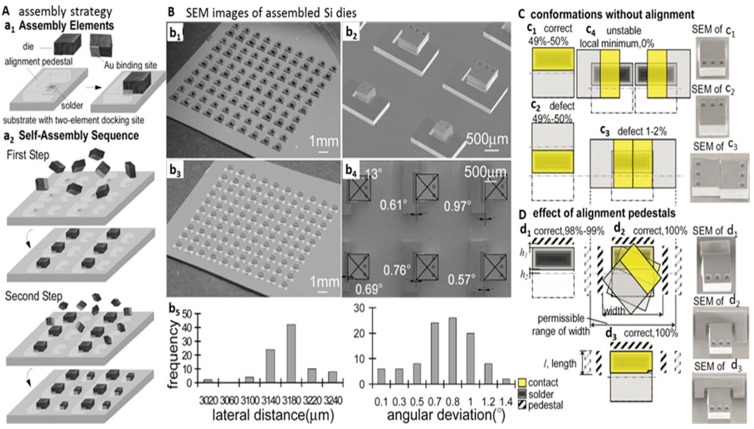
Strategy (**A**) results (**B**) and design rules (**C**) to assemble and connect chip-scale components with different dimensions, single-angular orientation, and contact-pad registration. (**a_1_**) Self-assembly elements depicting dies with a gold contact and two-element docking sites containing alignment pedestals and solder-coated areas. (**a_2_**) Two-step self-assembly sequence to populate the substrate with different components. The reduction of the interfacial free energy drives the assembly process into a stable position. (**b_1_**–**b_4_**) SEM images of silicon dies that have been assembled using a combination of alignment pedestals, solder-coated areas, and sequential batch transfer. 10 × 10 array that contains alternating rows of 900 µm and 500 µm dies that are surrounded by Si alignment pedestals (**b_1_**,**b_2_**) and SU-8 alignment pedestals (**b_3_**,**b_4_**) that was used to study the alignment accuracy. (**b_5_**) Histograms of the measured values. (**C**) Computer-aided design (CAD) approach that is used to eliminate defects and establish design rules together with experimental results. Rectangular receptor/binding site design that favors the 0° (**c_1_**) and 180° (**c_2_**) angular orientation along with predictions of defects (**c_3_**) and (**c_4_**). (**D**) Alignment-pedestal designs to eliminate defects. (**d_1_**) Single-pedestal designs with h1 ≤ h2 eliminate the 180° conformational overlap and yield the correct orientation in 98% of the cases. (**d_2_**,**d_3_**) Pedestals on the side eliminate the attachment of two components. *l* and *w* indicate the permissible range of the opening to ensure defect-free assembly. Reprinted with permission from [39].

Figure 3A presents an experimental strategy of assembling components with angular orientation control. The components were assembled using solder-coated areas that were partially surrounded by raised u-shaped alignment pedestals. Only correctly aligned components were captured and assembled; components with a deviation of more than ±90° were not assembled due to insufficient overlap between the component’s contact pad and the solder-coated binding site. Using optimized designs, we were able to obtain an angular orientation accuracy of 0.3° with a filling factor of 100%.

In the experiments, Si dies with dimensions of 900 μm × 900 μm × 500 μm and 500 μm × 500 μm × 500 μm were used in subsequent batch assembly processes. The assembly was performed in a glass vial filled with ethylene glycol at a temperature of 150 °C using solders with melting points of 47 °C and 138 °C. Figure 3B shows the result of the experiments. Subfigures b_1_ and b_2_ present the results with Si pedestals, whereas b_3_ and b_4_ present SU-8 pedestals. During the study, the authors observed that the width, length, and height of the alignment pedestals were major parameters to obtain high assembly rate and yield. Studying different conformations of the pedestals (Figure 3C), the authors developed CAD design rules to obtain maximum assembly rate with correct angular orientation.

## 4. Assembly of Microscopic Chips (10 μm–1 mm) at a Liquid-Liquid-Solid Interface

### 4.1. Chip Arrays Containing Isolated Si Chiplets

It can be noticed that all of the above-described techniques used components with sizes of several hundred microns. At this scale, the mass of the components is sufficiently high that gravity or acceleration (shaking) can be used to introduce or agitate the components during the assembly process. A challenge arises if the component size is reduced to the sub 100 μm range. Here gravity does not play an important role anymore. For assembling components at these dimensions, a new transport mechanism may be required, since gravity- or sedimentation-driven assembly processes [40] are exceedingly less effective. Knussel *et al.* reported an effective transport mechanism to assemble 10 μm–100 μm-sized chips [40]. The assembly took place in a conveyor belt-like fashion in a progressive linear front at a liquid–liquid–solid interface. The process was engineered in order to provide a stepwise reduction of interfacial free energy, which provided an energy cascade to (i) transport the components from a suspension to the interface (55 mJ/m^2^); (ii) pre-orient the components within the interface to face the right direction (90 mJ/m^2^); and (iii) assemble the micro-components at a predefined surface (400 mJ/m^2^). We tested a water–oil interface with SU-8 (20 μm × 20 μm × 10 μm) and Si (60 μm × 60 μm × 20 μm) components. One of the surfaces of the components was coated with gold and was treated to make it hydrophilic, and Si faces were treated to make them hydrophobic.

**Figure 4:**
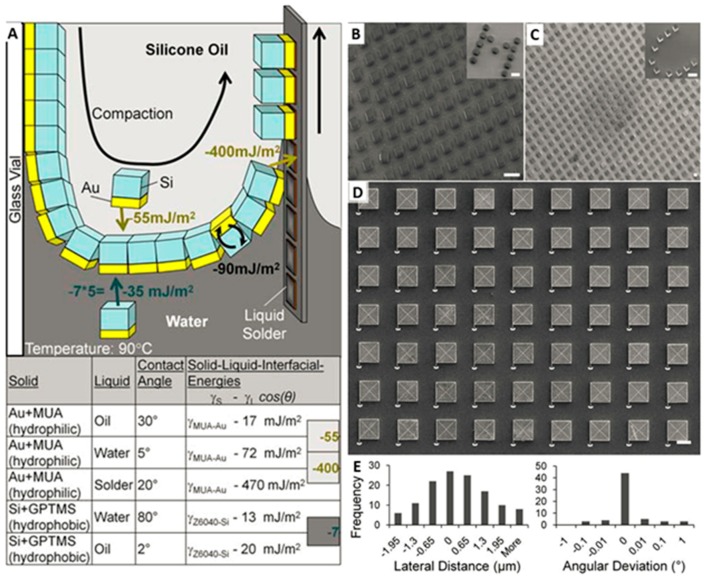
(**A**) Mechanism of surface tension directed self-assembly at a liquid–liquid–solid interface employing an energy cascade to (i) move components from a suspension to the interface, (ii) pre-orient the components within the interface to face in the right direction, and (iii) assemble the components on molten solder through dipping. The illustration depicts the situation for an oil–water interface and chiplets made out of Si (SU-8 is detailed in the main body), which carry an Au contact on one face. Depicted Au and Si surfaces are treated using hydrophilic mercaptoundecanoic acid (MUA) and hydrophobic 3-glycidoxypropyltrimethoxysilane (GPTMS) functional groups and yield the tabulated measured contact angles, calculated solid–liquid interfacial energies, and energy differences (gray boxes to the right) required to drive the assembly. The available area and curved shape of the interface cause the components to form a closely-packed 2D sheet. Upward motion of substrate yields a dynamic contact angle where the receding water layer becomes sufficiently thin for the gold to contact the solder. SEM images of SU-8 (**B**) and Si (**C**,**D**) chiplets assembling in regular arrays and arbitrary text patterns (insets). The overlaid CAD guides visible in (C, white lines) are used to measure variations in the center-to-center distance and angular orientation. (**E**) Histogram of measured variations. 40 µm scale bars. From [40], copyright (2004) National Academy of Sciences, USA.

Figure 4A illustrates the mechanism of surface tension directed self-assembly at a liquid–liquid–solid interface. In the process, the water–oil interface oriented the components in the right order, and a pre-solder-coated substrate was pulled upward at a speed of 30 mm/s. Correctly-oriented components were assembled on the substrate at the interface of the water–oil–solder-coated substrate. For the attachment process, the solder needed to be molten, which was achieved by heating the oil to 95°C. To achieve full coverage, several passes were required. Figure 4B–D present the result of the assembly. The prototype system achieved a very high throughput: 62,000 components were assembled and connected electrically and mechanically in three minutes. The spatial accuracy of the assembled components was 0.9 µm lateral with only a 0.14° angular deviation (Figure 4E), which exceeded previously reported results.

### 4.2. Self-Tiling—Chip Arrays containing Si-PIN Diodes

**Figure 5:**
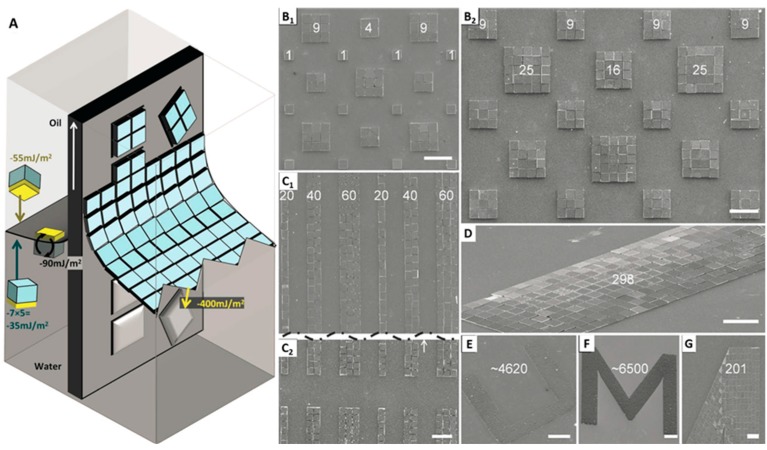
Strategy to assemble and connect chip-scale components with different dimensions, single-angular orientation, and contact-pad registration. (**A**) The available area and curved shape of the interface cause the components to form a closely packed 2D raft. Upward motion of the substrate yields a dynamic contact angle where the receding water layer becomes sufficiently thin for the gold to contact the solder domain, allowing the sections of the raft to transfer to the solder domain. Patterned transfer and self-assembly on molten solder is favored by 400 mJ/m^2^ within this layer. SEM images of tiled domains of different size: Square domains with room for 1, 4, 9, 16, and 25 silicon tiles, 60 μm on a side. 5 mm-long linear domains measuring 1, 2, and 3 component widths wide: (**C_1_**) center and (**C_2_**) end region. Rectangular domain with 300 Si-tiles (**D**), large letter-shaped domain for thousands of 20 µm-wide SU-8 tiles (**E**,**F**). (**G**) Grain boundary in asymmetric elbow section of letter-shaped regions. Scale bars 180 µm for (**A**–**G**) and 440 µm for (**E**,**F**). Reprinted with permission from [41].

The previously reported assembly process was subsequently extended to support packing of small semiconductor tiles to produce functional photovoltaic cells [41]. As shown in Figure 5A, assembly took place at the water–oil–solid interface on a flexible substrate which was pre-coated with solder in a defined pattern to accommodate a different integer number of components. When the sample is pulled upward through the water–oil interface with a speed of 30 mm/s, the Au-coated component side gets into contact with molten solder with a reduced contact angle. This causes a reduction of the surface energy of the molten solder bumps, and consequently the assembly of components. The substrate domains were patterned in different dimensions to accommodate different numbers of dies. Figure 5B_1_–G are the images of the tiled dies with different domains. Presented results show that the tiling was possible from a single domain to a large domain where several thousands of components were accommodated in tiling fashion without any intermediate gap. The largest domain contained over 6500 tiles closely packed with less than 3% vacancies. However, crystallographic defects were observed where crystal fronts merged in rounded region and in elbows (Figure 5G).

## 5. Reel-to-Reel Assembly

### 5.1. Applied to Solid State Lighting

**Figure 6:**
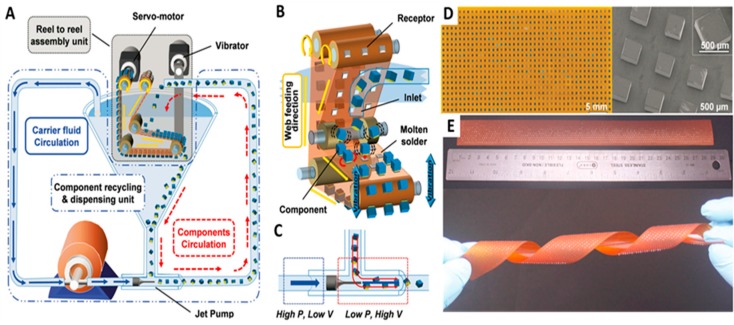
Automated reel-to-reel (RTR) fluidic self-assembly scheme. (**A**) Overview depicting (i) component dispensing on the basis of liquid flow and gravity, which transports the components to empty self-assembly sites (receptors) on a continuously advancing web, (ii) where the components attach at predetermined locations on the basis of surface tension directed self-assembly, and (iii) where recycling of access components is achieved on the basis of gravity at the turning point of the web before they are transported upward on the basis of a fluid drag inside a sufficiently-small-diameter channel. Mechanical up and down motion of the web is used to induce liquid flow and shear forces to dispense and agitate the components as well as to aid in the removal of access component in the region of the upside-down-oriented web. (**B**) Schematic depicting the details of surface tension directed self-assembly using molten-solder-bump-based-receptors to capture and connect to the metal contacts on the chips. (**C**) A jet pump-based principle is used to achieve recirculation of the chips without passing the components through a mechanical pump where they would get damaged. A preferred location of the jet pump is at the bottom of the slanted funnel where access components arrive on the basis of gravity to be picked up by a high velocity directional jet. (**D**) Periodic assembly onto 1.1 mm pitched square lattice points, 820 chips, 6 defects. Photograph and (**E**) Overview depicting continuous assembly onto a 2 cm wide and 29 cm long assembly region. Reprinted with permission from [42].

In terms of assembly rate, yield, and device dimensions, the self-assembly processes discussed so far are very promising. In principle, the reported methods should be scalable to enable large volume production. This should be a research theme going forward. In our opinion this is critically important and will require an increased effort towards machine designs, tests, verification of scalability, and more discussions on what methods are most suitable for real-world applications. First designs are being considered [42,50,51,52]. Park *et al.* reported a first implementation of an automated reel-to-reel (RTR) fluidic self-assembly machine [42]. Figure 6A illustrates the schematic of the referred RTR machine. The platform had two main parts: an assembly unit shown in gray, and a component recycling and dispensing unit, shown in blue. The assembly unit consists of a servo motor, vibrator, rollers, and polyimide; this unit controls relevant parameters such as agitation, web moving speed, and tension of the web. The dispensing unit used a jet pump to cycle the components. Figure 6B describes the attachment process of the chips onto the advancing substrate. Like in previous reports, the substrate was patterned solder-coated. The solder-coated sites act as receptors for the components and provide electrical and mechanical connections to the substrate. The assembly took place inside water that was heated to 80 °C in order to melt the solder. A nozzle was used to transport the components on the assembly web, which was moving at a velocity of 10 m/h. The components arrive on the web by gravity-driven sedimentation (Figure 6C).

Figure 6D,E presents the results of the assembly process using the RTR machine. During the experiments, components with different dimensions were studied. Experimental results show high assembly rate and yield with 99.3% to 99.8%. Using the prototype machine with a single nozzle and 2.5 cm wide assembly web, 15,000 chips were assembled in one hour, which exceeds the throughput of the fastest robotic pick-and-place machine reported so far. This rate could be increased linearly by increasing the number of nozzles and web width. For example, using a 25 cm web, the assembly rate would be 150,000 chips per hour.

### 5.2. Applied to Stretchable Electronics

The presented results assembled the electronic objects either on rigid or flexible substrates. There is a recently-emerging field of stretchable electronics, which requires assembly and electrical connection on soft and rubber-like substrates. Alignment and registration on soft and stretchable materials are challenging, and robotic pick-and-place and transfer are difficult to be carried out for small size chips on such substrates. A process of fluidic-self-assembly that overcomes alignment issues could potentially be used as an alternative.

**Figure 7:**
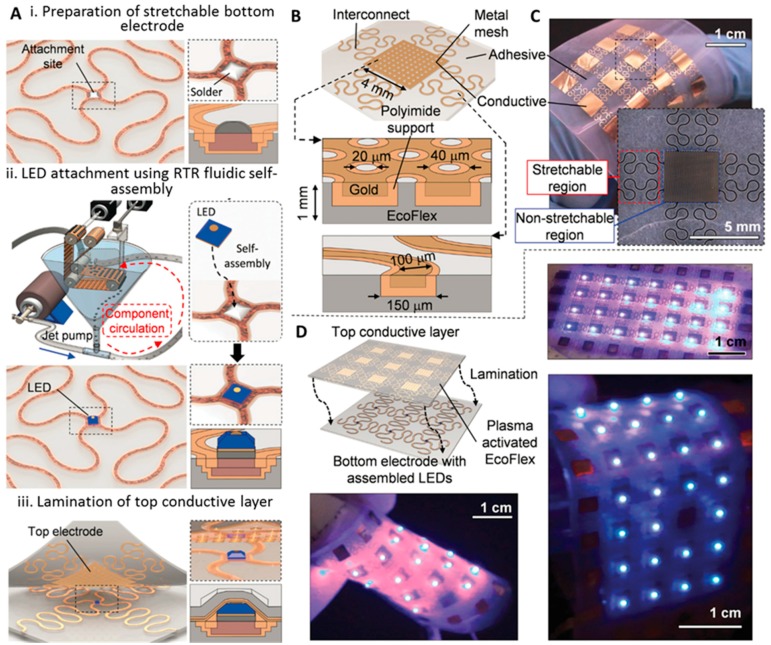
(**A**) Overview of the three basic steps used to fabricate a rubber-like solid-state lighting module: (i) preparation of a stretchable bottom electrode with solder-coated attachment site; (ii) the attachment of the LEDs using solder-directed fluidic self-assembly; and (iii) the lamination of a stretchable top conductive layer. (**B**) Schematics and (**C**) photographs describing the design, fabrication, and application of the stretchable top conductive lamination layer next to results of a completed 2 mm thin rubber-like module under test. (B) The designed laminate is a composite made up of: (i) conductive and (ii) adhesive, as well as (iii) stretchable (meander), and (iv) non-stretchable (metal mesh) regions; oxygen plasma-activated EcoFlex acts as an adhesive during the lamination process. (**C**) Photographs of the realized stretchable top conductive laminate. (**D**) The lamination is presently done by hand; as a consequence, we used a 4 mm × 4 mm metal mesh to tolerate a 2 mm misalignment with respect to the center contact of the LED. Outside this limit, the top contact will not be contacted. The bottom photograph shows a completed lighting module under various levels of deformation. Reprinted with permission from [49].

Park *et al.* reported first tests and the realization of a millimeter thin and rubber-like solid state lighting module fabricated using a roll-to-roll self-assembly machine, as described above [53]. The assembly process followed the same procedure as described in [42]. Figure 7A presents the overview of the basic steps of fabrication and assembly: (i) fabrication of the bottom electrode; (ii) assembly of LEDs; and (iii) lamination of the top electrode. The bottom electrode was designed as meander-shaped structure to tolerate the stress during stretching. It had two main components: a stretchable region, and a non-stretchable region for assembly of the rigid component. Experimentally, the bottom electrode alone could be stretched up to 220% of the original length without breaking the electrical contact to the assembled component. A cured silicone (Ecoflex) foil was used as a stretchable substrate. LEDs (300 μm × 300 μm × 150 μm) with bottom (large square) and top contact (small circle) pads were used. Assembly was performed using the RTR machine on Ecoflex with pre-coated solder on the receptor sites. The top contact was made to the components by lamination of the top electrode. The electrodes separately had a stretchability of 220%, the stretchability of the entire system after lamination of both electrodes was reduced to 170%.

## 6. Conclusions

In conclusion, processes of directed self-assembly have features which makes them attractive and powerful from a manufacturing point of view. They enable assembly of microscopic objects that goes far beyond the capabilities of robotic pick and place. For example, integration of chip sizes under 300 µm is challenged for the pick and place. Furthermore, applications which require assembly of chips on various substrates, especially on non-planar topologies are restricted, considering the current robotic chip assembly platforms. Moreover, considering assembly in large numbers, high volume and density over large areas at low cost is also challenged due to the serial nature of pick and place capabilities. The solid state lighting we focused on is only one of those fields.

Going forward, research can go in many directions. First, a good area is the assembly of microscopic chips at the 1–100 µm length scales. Here, robotic methods are highly challenged and a directed self-assembly process is potentially the only effective solution to redistribute the chips. Particularly on the microscopic length scale, knowledge needs to be increased with respect to the type of component introduction and agitation that will work to maintain acceptable levels of yield. Second, the number and minimal size of interconnects per chip needs to be improved. For example, a flip chip self-assembly process which forms a larger number of interconnects (at least two, and preferably more) continues to be highly desirable. If this cannot be achieved, alternative solutions should be studied. One alternative is to combine fluidic self-assembly with transfer. Fluidic self-assembly would be used to re-distribute the chips, which are subsequently transferred onto a final surface enabling flip-chip orientation. Another challenge is the temperature stability of the interconnects. The low melting point solder limits the final operating temperature. Two potential solutions are known. The first solution would involve flip chip transfer processes that decouple the self-assembly process from the interconnection process. Self-assembly is conducted first using one face of the chips to distribute the chips over a desired area onto an intermediate carrier. The intermediate carrier is flipped and aligned with the target substrate to transfer the components in a single step. The transfer step can involve a higher melting point solder and increase the melting point. Moreover, an increase in density and number of interconnects can be anticipated using this solution.

The second solution would involve a solder stack with a low melting point outer shell to enable assembly at low temperature [42,50,51,52]. After assembly, a short higher temperature reflow step could be used to form an alloy to increase the melting point of the interconnects.

Considering the current state of the art, an immediate application can be found in the field of solid state lighting. This application requires the distribution of light sources. A distribution of other devices can also be interesting. For example, the distribution of transistors and switches can be used to produce an active matrix array for sensing or emitting structures.
